# Smart Online Coffee Roasting Process Control: Modelling Coffee Roast Degree and Brew Antioxidant Capacity for Real-Time Prediction by Resonance-Enhanced Multi-Photon Ionization Mass Spectrometric (REMPI-TOFMS) Monitoring of Roast Gases

**DOI:** 10.3390/foods9050627

**Published:** 2020-05-14

**Authors:** Hendryk Czech, Jan Heide, Sven Ehlert, Thomas Koziorowski, Ralf Zimmermann

**Affiliations:** 1 Joint Mass Spectrometry Centre, Chair of Analytical Chemistry, Institute of Chemistry, University of Rostock, Dr.-Lorenz-Weg 2, 18059 Rostock, Germany; jan.heide@uni-rostock.de (J.H.); ralf.zimmermann@uni-rostock.de (R.Z.); 2 Joint Mass Spectrometry Centre, Cooperation Group “Comprehensive Molecular Analytics”, Helmholtz Zentrum München-German Research Center for Environmental Health GmbH, Gmunder Str. 37, 81379 München, Germany; 3 Photonion GmbH, Hagenower Str. 73, 19061 Schwerin, Germany; sven.ehlert@uni-rostock.de; 4 Department Life, Light & Matter, University of Rostock, Albert-Einstein-Straße 25, 18059 Rostock, Germany; 5 PROBAT-Werke von Gimborn Maschinenfabrik GmbH, Reeser Str. 94, 46446 Emmerich am Rhein, Germany; T.Koziorowski@probat.com

**Keywords:** photoionization mass spectrometry, polyphenols, process control, real-time monitoring, chemometrics, Arabica coffee

## Abstract

Process control with high time resolution is essential to maintain high product quality in coffee roasting. However, analytical techniques for quality assurance or measurements of desired coffee properties are often labor-intensive and can only be conducted after dropping the coffee beans. Resonance-enhanced multi-photon ionization time-of-flight mass spectrometry (REMPI-TOFMS) at 248 nm and 266 nm was applied to analyze the composition of the roast gas from small-scale Arabica coffee roasting. Coffee beans were dropped after different roasting times, ground and analyzed by Colorette to obtain the roast degree. Additionally, the antioxidant capacity of the coffee brew was determined by Folin–Ciocalteu (FC) assay. Models for the prediction of Colorette and FC values from REMPI mass spectra were constructed by partial least squares (PLS) regression. REMPI-TOFMS enables the prediction of Colorette values with a root-mean-square error in prediction (RMSE_P_) below 5 for both wavelengths. FC values could be predicted using REMPI at 248 nm with an RMSE_P_ of 80.3 gallic acid equivalents (GA-eq) mg L^−1^, while REMPI at 266 nm resulted in RMSE_P_ of 151 GA-eq mg L^−1^. Finally, the prediction of Colorette and FC value at 5 s time resolution were demonstrated with online measurements.

## 1. Introduction

Monitoring and control of industrial food processing is essential to maintain highest quality standards and reproducibility of the product. The production of one of the world’s most popular beverage coffee involves several steps from harvesting the fruit of the coffee bush to the final coffee cup [1]. After green bean processing, roasting of the coffee beans is in the focus of the production chain, which is often regarded rather as art than science. By analyzing the composition of volatile organic compounds (VOC) in the roast gas, valuable information about the state of the roast and underlying processes can be obtained. In this connection, the choice of the analytical tool is crucial because of the trade-off between time resolution and chemical specificity: Individual isomer-resolved VOC in the headspace of ground coffee can be resolved by chromatographic techniques [2,3], however, the time resolution is not sufficient to change roast conditions based on the analytical result. On the contrary, direct measurement techniques, such as near-infrared spectroscopy (NIR) [4] or time-of-flight mass spectrometry (TOFMS) with proton-trans reaction (PTR) [5,6], single-photon ionization (SPI) [7,8,9] and resonance-enhanced multi-photon ionization (REMPI) [8,9,10] as soft ionization techniques, are able to illustrate the temporal evolution of VOC released from Maillard, Strecker, caramelization and pyrolysis reactions. REMPI-TOFMS at 266 nm, which is a selective analytical technique for aromatic compounds, firstly monitored the thermal degradation of chlorogenic acids through detection of phenolic species by an online measurement [11]. Higher chemical specificity, but still satisfactory time resolution, was achieved by coupling TOFMS to fast separation techniques, such as ion mobility spectrometry [12] or ultra-fast gas chromatography [13], or by wavelength selection in REMPI [9].

In addition to fundamental investigations of coffee roasting, some coffee properties, such as coffee bean color or antioxidant capacity, are of great interest in quality control or beneficial for human health. The coffee bean color is simply measured by red light and/or NIR reflectance of a coffee sample and can be expressed in different metrics. While CIELAB or Hunter-L span a three-dimensional color space, simpler metrics, such as Agtron or Colorette, are restricted to a single value describing the roast degree.

Coffee beverages are known to contain substantial amounts of antioxidants, such as polyphenolic acids, stilbenes, lignans, tannins, and flavonoids, depending on the coffee cultivar, geographic origin and roast conditions [14]. In particular, these polyphenolic species gained attention in food research because foods rich in polyphenolics have been associated with the prevention of inflammation-related diseases, cancer, cardiovascular diseases, neurodegenerative diseases, diabetes, obesity, and osteoporosis [15,16,17]. Although polyphenols and their metabolites may directly scavenge free radicals, negligible importance was attributed to this mode of action in the human body [18]. More likely, their ability to act as chelating agents for redox active metals and modulatory actions are the key mechanisms. For example, polyphenols may affect the enzymatic activity of the gut microbiota or act on protein kinase and lipid kinase signaling pathway, preventing from glucose intolerance and diabetes [19,20]. Even in healthy men, a reduction in polyphenol-rich food may affect vascular biomarkers [21]. Therefore, product optimization toward healthy foodstuffs or conversion into so-called functional foods may be beneficial for human health.

In foodstuffs, antioxidants are often measured by electrochemical biosensors [22] or antioxidant assays, which can be classified by the underlying mechanisms in electron-transfer and hydrogen-atom-transfer antioxidant assays [23]. However, these techniques can only be used after dropping, grinding and brewing the roasted beans and may be also involve labor-intensive sample preparation for the analytical measurement, which also holds for bean color determination. On that account, chemometric approaches such as partial least square (PLS) regression or principal component analysis were applied on data from analytical instruments with high time resolution. Consequently, coffee properties of interest, such as titratable acidity, roast degree, antioxidant capacitym or content of sucrose, were made accessible during the entire roasting process [6,24,25,26,27].

This study continued the work of Heide et al. [28], who demonstrated the feasibility of using SPI-TOFMS for the online prediction of roast degree and antioxidant capacity, with REMPI-TOFMS for small-scale coffee roasting. PLS regression models for roast degree and antioxidant capacity by means of Colorette and Folin–Ciocalteu (FC) assay were generated with data from REMPI-TOFMS, which was conducted with two established industrial laser systems. The resulting four prediction models were extensively characterized, compared with each other and literature models, and finally applied to online REMPI-TOFMS data for real-time prediction.

## 2. Materials and Methods

### 2.1. Drum Roaster and Coffee Beans

**Figure 1:**
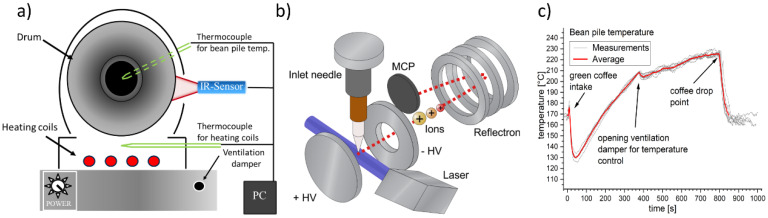
(**a**) Roast experiments for batch sizes of 100 g were conducted with a coffee drum roaster, which was electrically heated and equipped with a thermocouple to determine the bean pile temperature. (**b**) The instrumental setup consists of an Nd:YAG laser and non-linear optics to produce 266 nm UV-radiation or a KrF excimer laser providing 248 nm radiation for resonance-enhanced multi-photon ionization (REMPI). Ions were separated and detected by a reflectron time of flight mass spectrometer (TOFMS), which allows monitoring of the roasting off-gas composition down to subsecond time resolution. (**c**) The bean pile temperature shows a typical profile for drum roasters, including a temperature drop after filling and rebound. The second smaller temperature drop is caused by increased air flow from opening of the damper. The grey lines represent individual roasts of approximately 14 min.

Green Arabica coffee beans (*Coffea Arabica*) from Colombia were roasted with an electrically heated single drum roaster (*PRE 1Z*, PROBAT-Werke von Gimborn Maschinenfabrik GmbH, Emmerich am Rhein, Germany) having approximately 100 g batch size. An integrated temperature readout and an infrared temperature sensor was used to monitor the bean pile temperature inside the inner drum and the outer wall temperature, respectively (Figure 1a). In particular, the temperature during the filling of the coffee beans has been shown to be crucial for roasting reproducibility.

In total, 84 roasts with REMPI-TOFMS at 248 nm and 69 roasts with REMPI-TOFMS at 266 nm were performed (Figure 1b). All roasts started with an outer drum temperature of (180 ± 2) °C, which is equal to setting the power consumption of the roaster to (480 ± 20) W. In order to avoid too steep ascent in bean pile temperature and too fast aroma development, the damper was opened after approximately 6 min causing a higher air flow. The resulting roasting profile and its variation is depicted in Figure 1c. However, it should not be compared to other roasting profiles regarding the absolute temperature because of the influence of the position of the thermocouple.

### 2.2. Resonance-Enhanced Multi-Photon Ionization Time-of-Flight Mass Spectrometry (REMPI-TOFMS)

Compounds of the roast gas were sampled from the roaster through a heated deactivated fused silica capillary (heated to 250 °C, inner diameter of 200 µm, length of 2 m) and analyzed by resonance-enhanced multi-photon ionization time-of-flight mass spectrometry (REMPI-TOFMS) either at 248 nm or at 266 nm (Figure 1c). First, a compound of the roast gas absorbs an UV-photon and becomes excited. If the lifetime of the excited state is sufficiently long, the absorption of a second UV-photon may exceed the energy barrier of ionization. Since the energy of two UV-photons is only 9.32 eV and 10.0 eV for 266 nm and 248 nm, respectively, the transferred excess energy on the molecule is small, turning REMPI into a soft ionization technique. The existence of the excited state connects the ion yield with UV spectroscopy and drives the wavelength-dependent selectivity of REMPI [29]. At 266 nm, almost exclusively aromatic compounds can be ionized, while at 248 nm, also aliphatic amines become accessible. Generally, main components of the roast gas, including nitrogen, oxygen, water, carbon dioxide, and carbon monoxide cannot be ionized due to their too high ionization energy.

UV radiation was either provided by a KrF laser (*PhotonEx*, Photonion GmbH, Schwerin, Germany; operated at 200 Hz repetition rate, maximum pulse energy at 248 nm of 6 mJ, pulse duration 5−10 ns) for 248 nm or by the fourth harmonic generation of a Nd:YAG laser (*Big Sky Ultra*, Quantel, Les Ulis, France; 10 Hz repetition rate; power density at fundamental wavelength 1064 nm of 7·10^6^ W cm^−2^, maximum pulse energy at 266 nm of 4 mJ, pulse duration 11 ns) for 266 nm radiation. Both lasers types are relatively robust and frequently used in industrial applications and process control [29]. The generated ions were separated according to their mass-to-charge by a TOFMS (*compact time-of-flight mass spectrometer II*, Stefan Kaesdorf Geräte für Forschung und Industrie, München, Germany) and detected by a chevron plate (Burle Electro-Optics Inc., Lancaster, PA, USA). The integrated photoionization mass spectrometer system for process analysis was developed by University of Rostock and Photonion GmbH (*PhotoTOF*, Photonion GmbH, Schwerin, Germany).

### 2.3. Measurmeent of Bean Color by Colorette

The color of the roasted coffee beans, i.e., the roast degree, was determined by first grinding (Sette 270, Baratza LLC, Seattle, WA, USA) and subsequent measurement of the Colorette color value (Colorette 3b, PROBAT-Werke von Gimborn Maschinenfabrik GmbH, Emmerich am Rhein, Germany). Colorette values are based on reflectance measurements of the beans at red and near-infrared light. The result is a dimensionless value calibrated from 0 to 200, with decreasing values toward darker bean colors. Values between 150 and 60 are typical for commercial coffee products, exceptionally down to 35.

### 2.4. Measurement of Coffee Brew Antioxidant Capacity by Folin–Ciocalteu (FC) Assay

The antioxidant capacity of the coffee brew was analyzed by Folin–Ciocalteu (FC) assay, which is known to be in particular sensitive for phenolic species, but gives distinctly lower responses for other relevant classes of antioxidants, such as melanoidins [30]. Therefore, also “total phenolic content” (TPC) can be found in the literature as a term for the result of the FC assay analysis.

First, 12 g of ground coffee was brewed with 200 ml of hot water (82 ± 1) °C and let steep for 2 min in a French press. Subsequently, the coffee brew passed a paper filter with a maximum pore size of 2 µm. The filtrate was diluted by a factor of 50, set to pH of approximately 10 by adding sodium carbonate (anhydrous sodium carbonate, purity > 99%, Fluka Chemie GmbH, Buchs, Switzerland) and finally mixed with the FC reagent which contains phosphomolybdate and phosphotungstate (Merck KGaA, Darmstadt, Germany). If antioxidative compounds are present, a blue complex is formed from the reaction of the FC reagent and quantified with a photometer operated at a wavelength of 765 nm (Hach DR 3900, resolution of 1 nm, Düsseldorf, Germany). The photometer was calibrated with ten equidistant concentrations from 340 to 8500 mg L^−1^ of anhydrous gallic acid (purity > 98%, Merck KGaA, Darmstadt, Germany) dissolved in deionized water (electrical conductivity < 1 µS cm^−1^). The resulting calibration function had a coefficient of determination of 0.999 and a residual standard deviation of 106 mg L^−1^ [28].

### 2.5. Data Analysis

#### 2.5.1. Data Pretreatment

Data analysis was conducted using Matlab (R2018, MathWorks Inc., Natick, MA, USA) with statistic toolbox and PLS library (libPLS1.95) [31]. After conversion and binning of ion flight times into *m/z*, mass spectra were averaged to 5 s time resolution, baseline corrected, normalized to total intensity (L_1_-norm) for *m/z* up to 350 and mean centered. However, *m/z* from 1 to 58, *m/z* which show peak intensities below the limit of detection in more than 5% of the mass spectra, and *m/z* related to caffeine (*m/z* 193 to 197) were excluded from the data set before normalization. Due to the high selectivity and softness of the ionization, the first set of *m/z* below 59 can only contain noise, while caffeine was found to decrease the performance of the statistical models due to high variability between similar roast conditions.

#### 2.5.2. PLS Regression Modelling and Optimization

**Figure 2:**
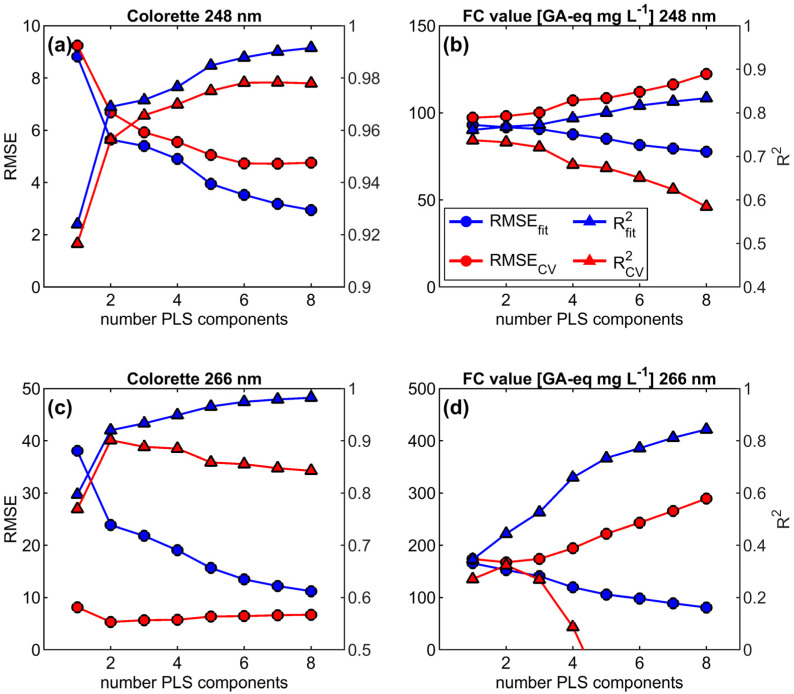
Results of explained variance from partial least squares (PLS) regression (R^2^_fit_, blue triangles), explained variance in Monte Carlo cross validation (R^2^_CV_, red triangles), root mean square error from PLS regression (root mean square error (RMSE)_fit_, blue circles) and root mean square error from Monte Carlo cross validation (RMSE_CV_, red circles) for the calibration data set over 8 PLS components for (**a**) Colorette values and REMPI at 248 nm, (**b**) Folin–Ciocalteu (FC) values and REMPI at 248 nm, (**c**) Colorette values and REMPI at 266 nm, and (**d**) FC values and REMPI at 266 nm. The selection of the optimal number of PLS components for modelling was based on the minimum RMSE_CV_ and maximum R^2^_CV_, respectively.

For the PLS regression models, the data sets of REMPI at 248 nm and 266 nm were first divided into a calibration (training) data set for initial model development and an external validation (test) data set to finally validate the model with a ratio of 4:1. Subsequently, the model development was started by fitting REMPI mass spectra of the training data to coffee properties Colorette and FC value using PLS regression [32] with an increasing number of PLS components. The resulting root mean square error (RMSE) and explained variance of the fit (R^2^) was then compared to RMSE and R^2^ from Monte Carlo cross validation (CV). For CV, 80% of the experiments in the calibration data set were randomly selected in order to calculate PLS regression coefficients and calculate RMSE from the prediction of the remaining 20% of the experiment. This procedure was repeated 1000 times each for the number of PLS components from 1 to 20. It is well known that PLS regression overfits by means of constantly decreasing RMSE (and vice versa increasing R^2^) with increasing number of PLS components. The selection of the optimal number of PLS components was initially based on the minimum RMSE in cross validation (Figure 2).

Because the discrepancy of RMSEs between the fit and the cross validation still indicates overfitting of the model, the variable selection technique competitive adaptive reweighted sampling (CARS) [33] was applied. CARS eliminates redundant variables using a survival-of-the-fittest algorithm leading to a decrease in RMSE and regularization of the model by reducing the number of PLS components in addition to the variable reduction. Similar to the initial model, the performance of the CARS-refined model was assessed by CV. With the new PLS regression model built from variables refined by CARS, RMSE and R^2^ of prediction (RMSE_P_ and R^2^_P_) were calculated using the external validation data set, giving a better estimate of the true RMSE and explained variance R^2^.

RMSE for fitting, CV and external validation (prediction) were calculated according to

(1) $$RMSE_{fit,CV,P}=\sqrt{\frac{\sum_{i=1}^{n}{\left(\hat{y_{i}}-y_{i}\right)}^{2}}{n}}$$

where $\hat{y_{i}}$ denotes the results of the regression, $y_{i}$ the measured coffee property and *n* the number of samples in the calibration or external validation data set, respectively. The explained variance for fitting and CV is calculated from the residual sum of squares (RSS) related to the total sum of squares (TSS) of the calibration data set

(2) $$R_{fit,CV}^{2}=1-\frac{RSS}{TSS}=1-\frac{\sum_{i=1}^{n}{\left(\hat{y_{i}}-y_{i}\right)}^{2}}{\sum_{i=1}^{n}{\left(\bar{y}-y_{i}\right)}^{2}}$$

with $\bar{y}$ being the average value of the measured coffee property. Furthermore, different metrics to calculate the explained variance in prediction has been evaluated in terms of its scaling invariance, invariance to RMSE_P_, correlation with RMSE_P_ and compliance with the ergodic principle [34]. Additionally, if the samples of the external data are not uniformly distributed over the range of the samples in the calibration data set, the comparison of R^2^ from external and calibration data set is biased [35]. Hence, the explained variance in prediction was determined using the formulae

(3) $$R_{P}^{2}=1-\frac{\sum_{j=1}^{n_{ext}}{\left(\hat{y_{j}}-y_{j}\right)}^{2}}{\sum_{i=1}^{n_{cal}}{\left(\bar{y}-y_{i}\right)}^{2}}=1-\frac{\frac{RSS}{n_{ext}}}{\frac{TSS}{n_{cal}}}$$

which refers to “*Q^2^_F3_*” in Todeschini et al. (2016) and overcomes the dependence on data distribution [34]. In contrast to formula (2), *RSS* is calculated from the external validation data set and normalized to the number of external samples (*n_ext_*), which is usually lower than the number of samples in the calibration data set (*n_cal_*).

## 3. Results and Discussion

### 3.1. Roast Degree and FC Value of Roasted Coffee Beans

**Figure 3:**
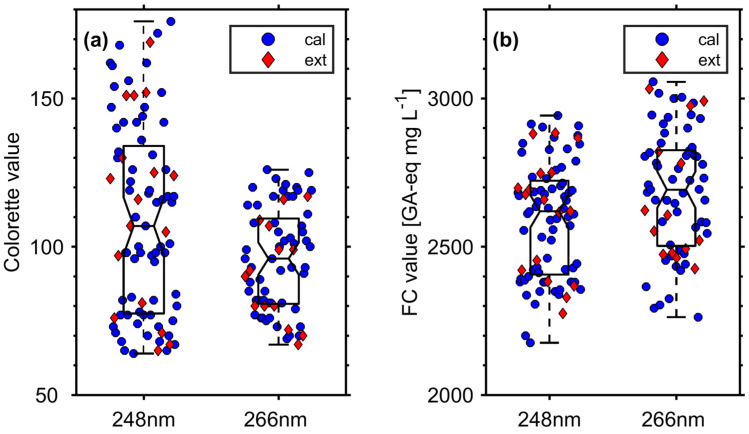
(**a**) Colorette and (**b**) FC values of single roast, divided by REMPI wavelength and their belonging to either the calibration (cal, blue circles) or test dataset (ext, red diamonds). The boxes show the 1st, 2nd (median), and 3rd quartile of the entire data set, while whiskers illustrate the full range (minimum and maximum) of the measurements. Since the notches of boxes in each subfigure do not overlap, the medians of the distribution from measurements at 248 nm and 266 nm are not significantly different at a confidence level of 95%.

The Colorette values of the roasted coffee beans lie in the range of commercial coffee products. Although the measurements conducted with REMPI at 248 nm span a broader range of Colorette values (177 to 62) than REMPI at 266 nm (125 to 64), the medians of the two Colorette value distributions do not differ significantly at 95% confidence since the notches of their boxplots overlap (Figure 3). Similarly, FC values from roasts with measurements using REMPI at 266 nm seems to be slightly higher than for REMPI at 248 nm. However, the medians of both FC value distribution do not differ significantly as well.

### 3.2. Components in the Roast Gas and Their Temporal Evolution

As previously described, REMPI at moderate laser intensities refers to a soft ionization technique, so most of the peaks in the mass spectra belong to molecular ions. The combination of the REMPI selectivity with results of previous studies involving chromatographic separation of roast gas compounds enables a tentative assignment of *m/z* to molecules (Table A1) [9,10].

**Figure 4:**
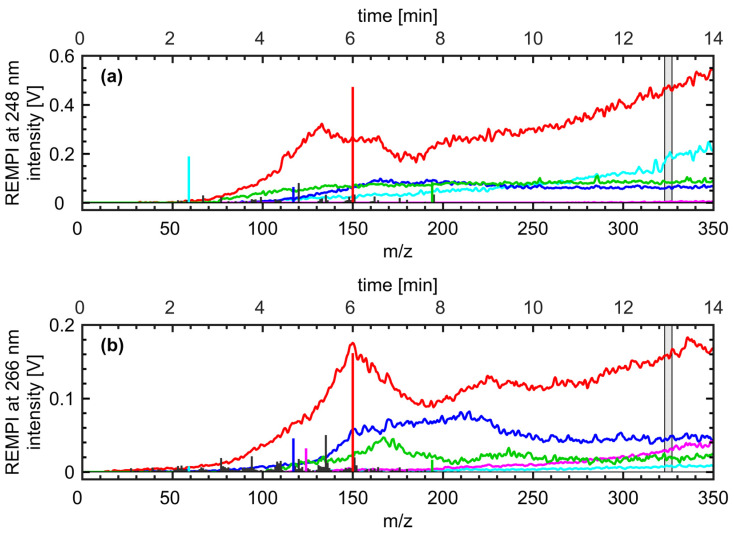
Combined average mass spectrum (vertical bars referring to *m/z*) at 13 min (average over grey shaded area) and mass traces of selected compounds (*m/z* 59 trimethylamine (cyan), *m/z* 117 indole (blue), *m/z* 124 guaiacol (magenta), *m/z* 150 vinylguaiacol (red), *m/z* 194 caffeine (green) referring to time) for REMPI-TOFMS measurements at (**a**) 248 nm and (**b**) 266 nm. In both mass spectra, vinylguaiacol denotes the base peak. However, REMPI at 248 nm is more sensitive than REMPI at 266 nm for many compounds in the coffee roast gas, in particular nitrogen-containing compounds. The REMPI selectivity at different wavelengths and consequence in coffee roast gas analysis are more detailed discussed in Czech et al. (2016) [9]. A list of detected ions with structure assignments and separated time traces can be found in Figure A1, Table A1, and Figure A2.

In both mass spectra of REMPI at 248 nm and 266 nm (Figure 4) from a roast of 14 min with a Colorette value of 73, the base peak appears at *m/z* 150, which refer to 4-vinylguaiacol, which is a decomposition product of ferulic acid. Ferulic acid is known as an early thermal decomposition product of chlorogenic acids, which accounts for approximately 1% of the green coffee bean weight. Further phenolic species appear at the *m/z* 124, 122, 110, 108, and 94, which can be assigned to guaiacol, dimethylphenol, benzenediol, methylphenol, and phenol, respectively. These compounds are increasingly released from the coffee beans as their thermal treatment of the coffee beans leads to further decomposition and consequently smaller decomposition products. Peaks at *m/z* 135, 107, and 77 arise from fragmentation of methoxyphenols and their derivatives as they easily decompose even at small excess energy [11]. However, the most abundant fragment *m/z* 135 from the elimination of a methyl group account only for less than 8% of the peak intensity at *m/z* 150. Further abundant peaks occur at *m/z* 194, 117 and 59, which can be assigned to the nitrogen-containing compounds caffeine, indole and C3-amines, respectively [9]. Although caffeine accounts for 1%−2% of the green Arabica coffee bean weight, its abundancy is limited because of its relatively low volatility and wavelength-dependent photoionization cross section. In contrast, indole has a higher volatility and is both contained in the green coffee bean as well as formed during the roasting from amino acids [36]. C3-amines are formed from the pyrolysis of amino acids [37] (p. 330) and an example of the altered selectivity of REMPI towards shorter wavelengths, enabling the detection of aliphatic compounds due to the sufficient lifetime of their excited electronic states. Overall, REMPI at 248 nm leads to higher intensities for most of the peaks, in particular for smaller nitrogen-containing compounds and five-ring heterocycles, such as furans and pyrroles, compared to REMPI at 266 nm, which is more selective for heterocycles and phenolic compounds with a higher degree of substituents at the aromatic ring.

It should be noted that these findings for the two wavelengths are strictly speaking only valid for this specific data sets because of slight differences in roast profiles on the one hand and different laser power and repetition rate (i.e., laser pulse energy), possible hotspots in the beam profile and beam shape on the other. The absolute ion yield, i.e. the ionization probability, depends on the squared intensity of the laser [38]. Despite of equal spot size, ion yields at 248 nm were higher, which is likely a consequence of the higher pulse energy (4 mJ) and repetition rate in operation (50 Hz) for the KrF laser compared to Nd:YAG (pulse energy: 2 mJ; repetition rate: 10 Hz). Furthermore, at moderate laser shot energies, the beam shape affects the amount of fragmentation and inhibits a direct comparison between REMPI mass spectra from Nd:YAG lasers with excimer lasers or optical parametric oscillators (OPO), even at the same wavelength.

### 3.3. PLS Prediction Models for Roast Degree (Colorette) and Antioxidant Capacity (FC Value)

#### 3.3.1. Figures of Merit

**Figure A3:**
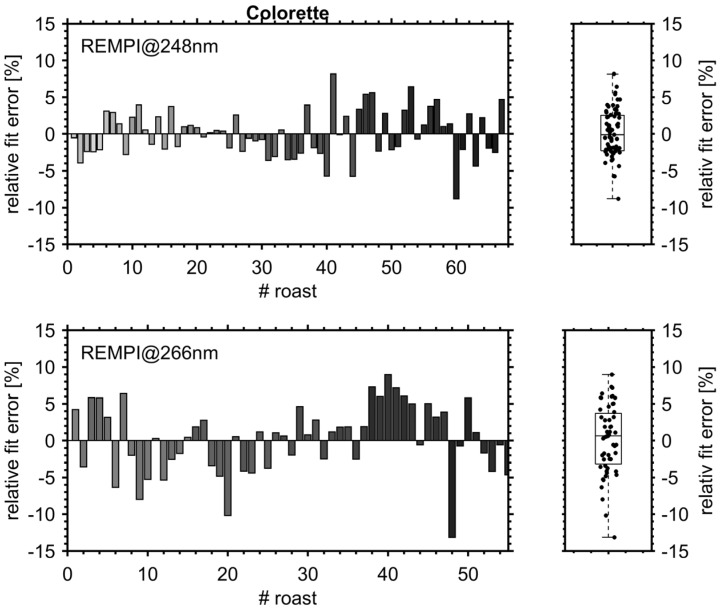
Relative fit error of individual roasts included in the calibration of the Colorette prediction models (**left**) and its summary in a boxplot (**right**) showing minimum, maximum, 1st, 2nd (median) and 3rd quartiles.

PLS regression models for the prediction of Colorette value reveal R^2^_P_ > 0.92 together with RMSE_P_ of 4.54 and 5.63 for 248 nm and 266 nm, respectively (Table 1). The residues between the measured and predicted Colorette values are normally distributed, analyzed with Shapiro–Wilk test at a significance level of 0.05. Also, with *p* > 0.49 a permutation test with 500,000 permutations did not indicate any first or second-degree polynomial trend in the regression residuals for any Colorette model [39]. The relative deviations for single measurements of the calibration data set have an interquartile range of ± 3% and ± 4%, respectively, and are illustrated in Figure A3. Moreover, RMSE and R^2^ from CV and external validation differ only slightly, indicating validity of the model. The range-error-ratio (RER) relates the range of coffee properties in the external validation set to the RMSE_P_:

(4) $$RER=\frac{y_{ext,max}-y_{ext,min}}{RMSE_{P}}$$

Good predictive models typically have a RER > 10, which is fulfilled for both Colorette value models with RER of 23 and 11 for REMPI at 248 nm and 266 nm, respectively. However, the distinctly lower RER for REMPI at 266 nm is likely caused by the smaller range of Colorette values since the RMSE_P_ is comparable between the two models. If the absolute RMSE_P_ is related to the range of the Colorette values in the calibration data set, the relative RMSE_P_ is obtained, which account for 4.0% and 8.0% for REMPI and 248 nm and 266 nm, respectively. Both RER and relative RMSE_P_ of this study are competitive with previously published RER and relative RMSE_P_ [25,28]. Another difference between the two Colorette value models arises from their complexity. While the model of REMPI at 248 nm contains 6 PLS components and make use of 16 different *m/z*, the model of REMPI at 266 nm is simpler having only 2 PLS components considering 10 different *m/z*. At similar prediction performance, the model of lower complexity should be preferred.

**Figure 5:**
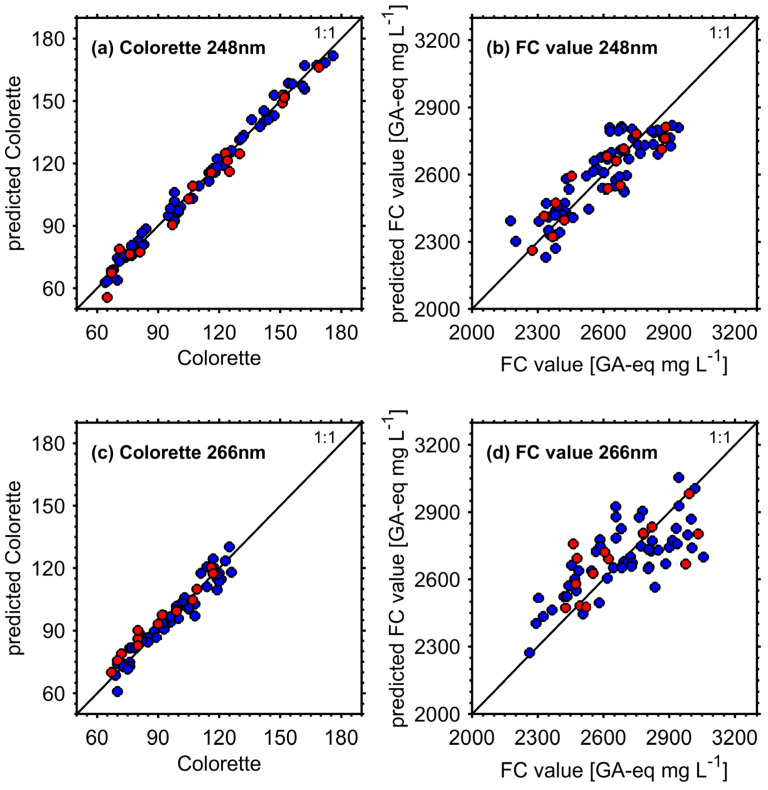
Relation between measured and predicted Colorette and FC values for REMPI at 248 nm (upper panels (**a**) and (**b**)) and REMPI at 266 nm (lower panels (**c**) and (**d**)). Blue circles refer to the calibration data set, red circles to the test set for external model validation.

**Figure A4:**
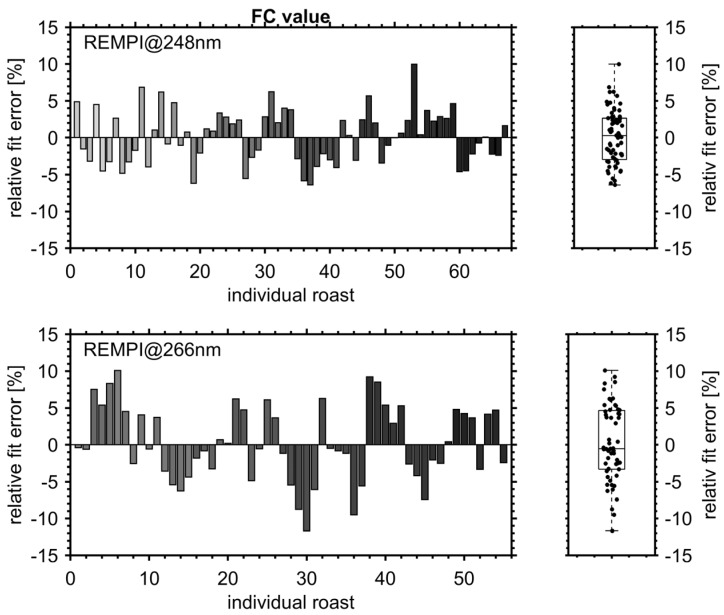
Relative fit error of individual roasts included in the calibration of the FC value prediction models (**left**) and its summary in a boxplot (**right**) showing minimum, maximum, 1st, 2nd (median), and 3rd quartiles.

With REMPI-TOFMS at both applied UV wavelengths, the prediction of FC values from the roast gas is possible, but with clearly lower predictive ability for REMPI at 266 nm. While with REMPI at 248 nm R^2^_P_ and RMSE_P_ of 0.822 and 80.3 GA-eq mg L^−1^ were still achieved, REMPI at 266 nm gave much poorer R^2^_P_ of 0.454 and RMSE_P_ of 151 GA-eq mg L^−1^. Compared to the residual standard deviation of 106 GA-eq mg L^−1^ for the FC value calibration [28], which is a quantity to describe the precision of the FC value measurement, the model using REMPI at 248 nm is competitive and may substitute the more labor-intensive and time-consuming FC value measurement. Despite good results for RMSE_P_, with 7.6 and 4.0 the RER of the FC value prediction models appear below 10. However, this might be caused by the relatively low range for FC values of only approximately 600 GA-eq mg L^−1^, which may be larger for other coffee or roasting variants, such as in Catelani et al. (2017) [26]. Furthermore, the predicted FC values are clearly more scattered round the 1:1 line in Figure 5 than the predicted Colorette values and apparently have a slope below unity. Nevertheless, both FC value prediction models passed the residual analysis using Shapiro–Wilk test, however, only the FC value prediction model with REMPI at 248 nm also passed a permutation test using second degree polynomial (with significance levels of 0.05 for both tests) [39]. Additionally, the models do not seem to suffer from a bias becasue the predicted values in both calibration and external validation data set are uniformly scattered around the 1:1 line. Relative deviations between measured and predicted FC values in the calibration data set have an interquartile range of ± 3% and ± 4% points, respectively, and are illustrated in Figure A4. 

Model refining by CARS did not reduce the number of PLS components further, but the number of variables down to eight and four for REMPI at 248 nm and 266 nm, respectively, which means that the model complexity is lower than for the prediction of Colorette value. Finally, the RMSE_P_ relative to the range of FC values account for 8% and 19% for REMPI and 248 nm and 266 nm, respectively. If RMSE_P_ is related to the mean FC value of the calibration data, 3.1% is obtained for REMPI at 248 nm, which appears close to the interday relative standard deviation of an FC assay coupled to size exclusion chromatography [40].

The often used ratio of performance to deviation or residual prediction deviation (RPD), which is calculated by

(5) $$RPD=\frac{RMSE_{P}}{\sqrt{\sigma_{ext}^{2}}}$$

where σ^2^_ext_ refers to the variance of the predicted quantity (here Colorette or FC value) in the external data set, is not presented as it makes use of the same metric as R^2^ does and depend on the underlying distribution of the data [41]. Therefore, often considered limits for good predictive models, such as RPD > 2, do not add information about the model.

**Figure A5:**
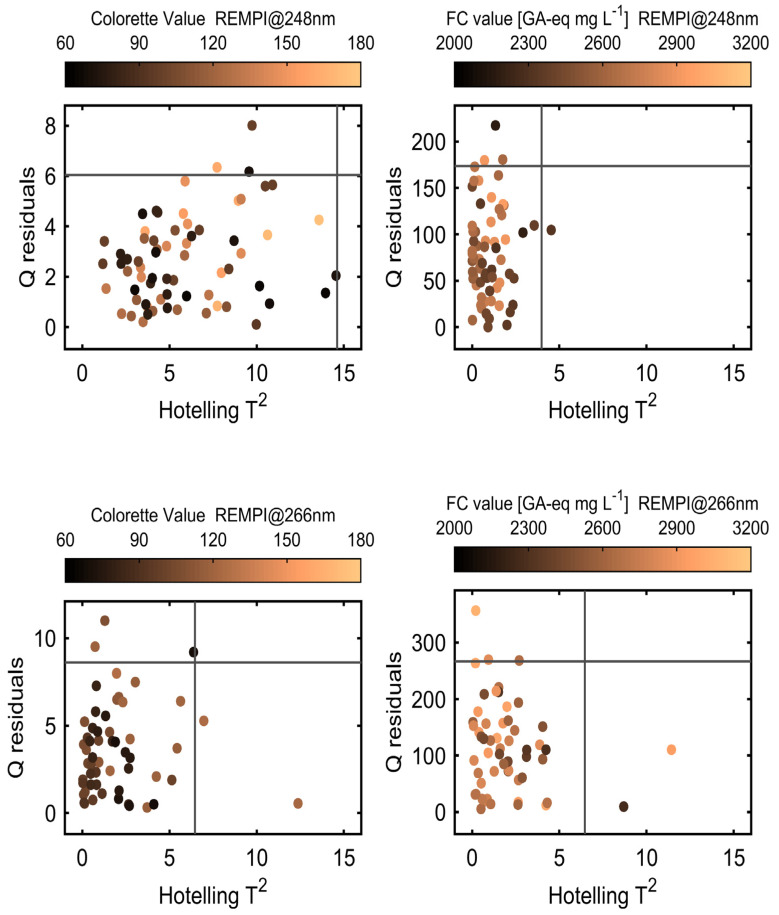
T^2^-Q diagnostic plots for outlier identification of samples in the calibration (training) data set. Larger Q appear for samples which are worse reproduced, while Hotelling’s T^2^ represents the influence of an individual calibration sample on the model. Vertical and horizontal lines denotes the 95% confidence limits. A calibration sample is regarded as outlier if it appears both outside the confidence limits for Q and Hotelling’s T^2^.

Outliers may significantly affect the model performance and also challenge its validity. However, such a sample could not be found in the data sets and in particular the models using data from REMPI at 248 nm show low scattering in the influence plot (Figure A5).

In addition to the model accuracies, sensitivity (SEN) defined as the change of signal with change of stimulus (e.g., concentration of an analyte), hence being the slope of the regression line in univariate calibration, is an analytical quantity of interest. Two approaches were selected from the literature and presented in Table 1. The first approach to calculate the sensitivity makes use of the regression coefficient vector *b*

(6) $$SEN_{b}=\frac{1}{\left\|b\right\|}$$

where ‖‖ refers to the L_2_-norm. Another approach derives *SEN* from net analyte signal (NAS) theory for inverse calibration models [42], which is further divided in the cases of known and unknown interferences of the NAS with different types of noise. From a mathematical point of view, the NAS refers to a vector orthogonal to the space spanned by analyte interferences. If the spectrum of the pure analyte is unknown, which is the most frequent case in multivariate calibration, the NAS v* can be obtained from the regression coefficients *b* for each mass spectrum *v* [43]

(7) $$v^{*}=b{\left(b^{T}b\right)}^{-1}b^{T}v$$

In the next step, the net sensitivity vector s* is calculated by relating the NAS v* to the measured concentration or property *c* (i.e., here Colorette or FC value) for the *ith* sample [44].

(8) $$s^{*}=\frac{v_{i}^{*}}{c_{i}}$$

Again, the L_2_-norm of s* gives the sensitivity *SEN_NAS’_*, while the sensitivity median of all samples in the calibration data set is presented in Table 2. *SEN_b_* and *SEN_NAS_* are for Colorette value models, but differ by a factor of three for the prediction of FC values. From *SEN*, the analytical sensitivity (*ASEN)* can be derived, taking the random noise *δx* of the measurement into account and giving a more meaningful quantity to compare different calibration models [45]:

(9) $$ASEN=\frac{SEN}{\delta x}.$$

For the results of *ASEN* in Table 2, *SEN_b_* was used. The inverse of *ASEN* (*ASEN^-1^*) provides information about the minimum difference of response measured between samples, which can be still distinguished by the method if the instrumental random noise is the only source of interference [45]. *δx* was determined from the first 10 s of the roast experiment, so the same gas sampling line was used as for the data from 7 to 14 min roast time. Clearly, the PLS regression models for Colorette values exhibit greater sensitivity; however, regarding *ASEN* and *ASEN^−1^*, REMPI at 266 nm shows worse performance, which agree with the figures of merit in Table 1.

The selectivity *SEL* presented in Table 2 denotes the proportion of the pure compound spectrum detected in a mixture. Strictly speaking, if the pure analyte spectrum is not available, *SEL* cannot be precisely defined [45]. However, a common method is to substitute the NAS vector v* as pure analyte spectrum and relate it to the spectrum of a mixture *v* by using their L_2_-norms [44]

(10) $$SEL=\frac{{\|v}^{*}\|}{\left\|v\right\|}\cdot 100$$

With 8.6% selectivity, the Colorette model for REMPI at 248 nm incorporate a distinctly lower fraction of the NAS vector than the other three models (Table 2). However, all *SEL* in REMPI PLS regression models appear in the range of previously published models for the online prediction of bean color [25] or FC value of coffee beans [26].

In the last part of the figures of merit, the limits of detection (LOD) obtained from different approaches are discussed (Table 3). A straightforward and common way to estimate the LOD from a multivariate calibration model is based on the RMSE_P_ [43] and calculated as:

(11) $$LOD_{3\times RMSE_{P}}=3\cdot RMSE_{P}.$$

Another approach aims to convert the multivariate into the univariate space by using the classical equation for univariate calibrations [46]:

(12) $$LOD_{pu}=\sqrt{3.3\cdot m_{pu}\cdot \left[\left(1+h_{0min}+\frac{1}{n_{cal}}\right)s_{r}^{2}\right]}.$$

For this pseudounivariate limit of detection (LOD_pu_), *m_pu_* denotes the slope of the regression from measured Colorette or FC values versus predicted Colorette or FC values, which is usually close to unity, *n_cal_* is the number of samples in the calibration data set and *s^2^_r_* the variance of the regression residuals. The minimum sample leverages *h_0min_* were calculated by

(13) $$h_{0min}=\frac{{\bar{y}}_{cal}^{2}}{\sum_{i=1}^{n_{cal}}y_{i}^{2}}$$

where *y_i_* denotes the centered calibration concentration [47]. In particular, models with REMPI data at 248 nm (Colorette: 6.34; FC value: 216 GA-eq mg L^−1^), but also REMPI at 266 nm (Colorette: 11.6; FC value: 687 GA-eq mg L^−1^) gave equal or lower LOD_pu_ than a recently published model based on SPI data (Colorette: 17; FC value: 687 GA-eq mg L^−1^) [28].

Similar to selectivity and sensitivity, the NAS concept can be deployed to calculate the LOD by incorporation of the instrumental noise *δ* and the sensitivity vector s* [48]:

(14) $$LOD_{NAS}=3\cdot \left(\frac{\delta }{{\|s}^{*}\|}\right)$$

From these three explicit methods, the first approach using Equation (11) led to the highest LOD for the Colorette prediction models, whereas the NAS approach Equation (13) was the lowest, spanning the range from 5.9 to 13.9. For the prediction of FC values, equations (11) and (12) gave comparable results at both REMPI wavelengths. In contrast, the NAS approach led to extreme low LOD at 248 nm and distinct higher LOD at 266 nm, which is caused by the sensitivities differing one order of magnitude between the two REMPI wavelengths (Table 2). However, when calculating the limit of quantification (LOQ) as the lowest concentration which can be determined with 95% confidence and 10% relative prediction error in a quantitative way from

(15) LOQ=3·LOD

it can be noticed that none of the measured values included in the models (Figure 3) appeared below the LOQ obtained from any of the presented methods. Converting LOD_NAS_ of this study from GA-eq mg L^−1^ into g kg^−1^, 0.595 g kg^−1^ at 248 nm and 10.2 g kg^−1^ at 266 nm were obtained, which is lower and similar to the result of 5.91 g kg^−1^ from Catelani et al. (2017) [26], respectively.

A recently published study pointed out that an extension of the definition of the LOD for the univariate case does not meets the complexity of multivariate calibration models and introduced a IUPAC-consistent LOD range instead of single values in order to take variations of the background composition into account [47]. The minimum and maximum LOD (LOD_min_, LOD_max_) is thereby obtained from the lowest and largest extrapolated leverages *h_0min_* and *h_0max_* from the calibration samples and calculated by

(16a) $$LOD_{min}=3.3\cdot \sqrt{SEN^{-2}var\left(x\right)+h_{0min}SEN^{-2}var\left(x\right)+h_{0min}SEN^{-2}var\left(y_{cal}\right)}$$

(16b) $$LOD_{max}=3.3\cdot \sqrt{SEN^{-2}var\left(x\right)+h_{0max}SEN^{-2}var\left(x\right)+h_{0max}SEN^{-2}var\left(y_{cal}\right)}$$

where *var(x)* refers to the variance in the instrumental signals and *var(y_cal_)* the variance in the calibration. For negligible variation in the calibration concentrations, i.e., for Colorette and FC value measurements, LOD_min_ approaches LOD_pu_. However, for higher variation in calibration concentrations, LOD_pu_ used to be higher than LOD_max_ [47]. Compared to the previously discussed three approaches for LOD, the LOD ranges are distinctly lower for Colorette, but similar for FC value prediction models. Above LOD_max_ and below LOD_min_, a signal can be interpreted similar to the univariate LOD by means of detected or not detected with a certain confidence, respectively. If a sample measurement appears within the LOD range, the sample-specific LOD (LOD_ss_) using its leverage must be calculated for a definite declaration [47]. For none of the samples, the predicted Colorette or FC value were lower than 3·LOD_ss_ (LOQ).

#### 3.3.2. Target Projection (TP) Loadings

Target projection (TP) loadings extract the relevant variation of the predictors, i.e., Colorette and FC values, in the descriptors, i.e., mass spectra, on a single vector [49]. In contrast to correlation-based extractions of relevant *m/z* such as selectivity ratio, TP loadings are based on covariance, hence accounting for differences in signal height between variables [50].

Due to the correlation between Colorette and FC values over the roasting [28], TP loadings for REMPI measurements at the same wavelength are similar. The TP loadings for Colorette and FC value have Pearson correlation coefficient of 0.980 at 248 nm and 0.598 at 266 nm. In contrast, correlations coefficients of only 0.408 and 0.002 were found for TP loadings of Colorette and FC values at different wavelengths (Table 4). Moreover, the number of variables after CARS is 50% lower for FC value models than for Colorette value models at the same REMPI wavelength. These findings emphasize again the difference in REMPI selectivity and the different performances of the prediction models.

**Figure 6:**
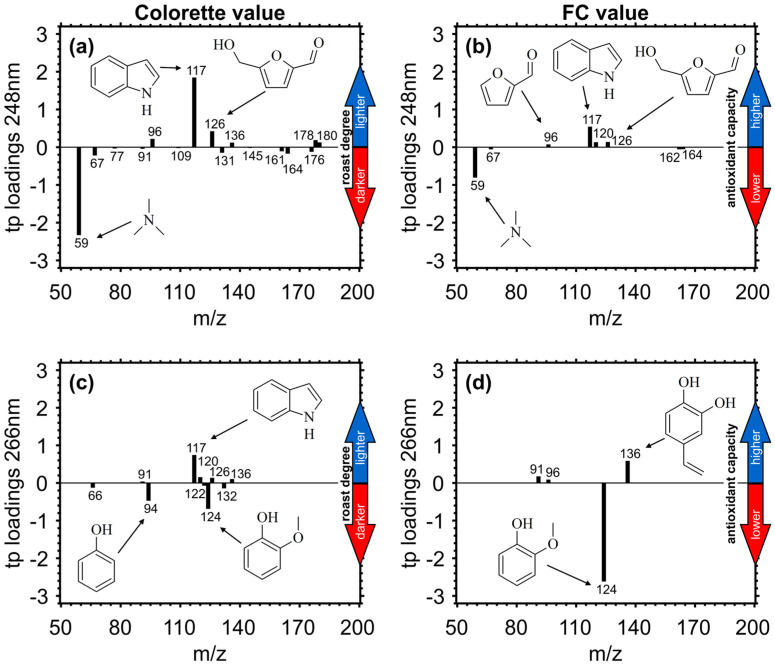
Target projection (TP) loadings as a metric of variable importance for prediction based on explained covariance for Colorette and FC values for REMPI measurements at 248 nm (upper panels (**a**) and (**b**)) and 266 nm (lower panels (**c**) and (**d**)). Positive TP loadings are more associated with higher Colorette and FC values, whereas negative TP loadings are more associated with lower Colorette and FC values. Structure assignments to *m/z* of TP loadings can be found in Table A1.

For the prediction of Colorette values with REMPI measurements at 248 nm, indole (m/z 117) and hydroxymethylfurfural (*m/z* 126) exhibit the highest TP loadings (Figure 6a). Indole may be formed from the thermal degradation of amino acids and shows its maximum abundance early during the roast. The class of furans, including compounds such as hydroxymethylfurfural (*m/z* 126), furfural (*m/z* 96) and difurfurylether (*m/z* 178), is a well-known product of the Maillard reaction. A phenolic species, such as vinyl-dihydroxybenzene (*m/z* 136), from the thermal degradation of chlorogenic acids, show positive TP loadings and was previously found associated with medium roasted coffee beans. Toward longer roasting times associated higher temperatures, nitrogen-containing species of low molecular weight, such as C3-amines (*m/z* 59) and pyrrole (*m/z* 67), play an important role for the prediction of lower Colorette values, i.e., darker bean color. However, the typical nitrogen-containing marker for dark roast, pyridine (m/z 79), does not appear in TP loadings because of its low photoionization cross section at 248 nm [9].

As shown by the high correlation coefficient, the TP loading vector for the prediction of FC values from REMPI at 248 nm measurements (Figure 6b) was very similar compared to TP loadings for the prediction of Colorette values and contained indole (*m/z* 117), the most prominent compound with positive loadings, and C3-amines (*m/z* 59) and pyrrole (m/z 67) with negative loadings. Despite alkaloids such as indole being known as effective antioxidants [51], they have likely low responses in FC assay measurements. Additionally, other m/z with positive TP loadings such as hydroxymethylfurfural (*m/z* 126) and furfural (*m/z* 96) have no significant antioxidant capacity [52]. Only 4-vinyl-phenol (*m/z* 120) refers to a phenolic antioxidant species [53], which is likely captured by FC assay. Hence, the prediction of FC values by REMPI-TOFMS at 248 nm is predominantly based on correlation rather than direct measurements of antioxidants.

At 266 nm, the positive TP loadings for the prediction of Colorette values are also dominated by indole (Figure 6c) despite its lower photoionization cross section than at 248 nm [54]. Compounds with lower positive TP loadings are hydroxymethylfurfural (*m/z* 126) and the phenolic species vinyl-dihydroxybenzene (*m/z* 136) and vinyl-phenol (*m/z* 120). Toward longer roasting times associated higher temperatures, substitutes at the aromatic ring become shorter, which agrees well with the most intense negative TP loadings for guaiacol (*m/z* 124) and phenol (m/z 94). For the prediction of FC values, vinyl-dihydroxybenzene (*m/z* 136) and guaiacol (*m/z* 120) account for more than 90% of the total absolute TP loadings (Figure 6d) and may be used to follow the roasting in a tentative manner. While at 248 nm REMPI is more sensitive for nitrogen-containing compounds, REMPI at 266 nm conversely generates higher ion yields from phenolic species [54].

#### 3.3.3. Toward Online Prediction of Colorette and FC Values in Real-Time

**Figure 7:**
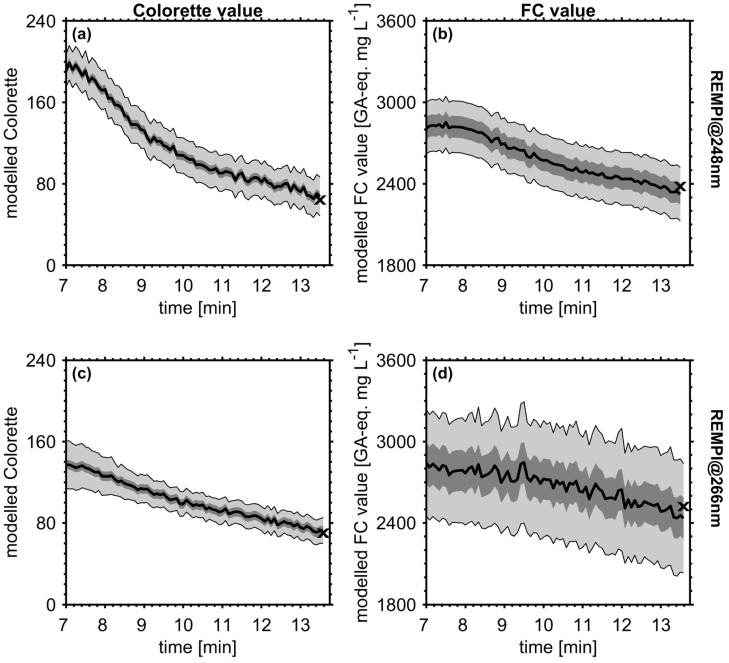
Application of PLS regression models for online prediction of Colorette (left) and FC value (right) within the range of calibration, enabling prediction of coffee properties in real-time (black line), with REMPI at 248 nm (upper panels (**a**) and (**b**)) and 266 nm (bottom panels (**c**) and (**d**)). Light grey-shaded area denotes the sample-specific prediction interval (PrI) with 95% confidence using equation (17), dark grey-shaded area denotes a constant error interval using RMSE_P_. The symbol (x) refers to the values measured by Colorette and FC assay.

The range of calibration samples covers approximately roasting time from 7 to 14 min, hence the model was applied on online data in order to enable the prediction of Colorette and FC values in real-time with 5 s time resolution (Figure 7). Similar to the study by Heide et al (2020) [28], both Colorette and FC values do not decrease linearly with ongoing roast time, emphasizing the need of tools for process control and monitoring. A crucial part in predicting properties of new samples is the estimation of the related uncertainty. For the comparison of model performance, the use of RMSE_P_ is appropriate; however, RMSE_P_ results in a constant error term and does not take into account the proximity of the sample to the model and the dimension of the predicted value. In other words, samples in the model center have lower prediction uncertainty than samples at the model periphery and larger values are associated with lager absolute errors. On that account, the dynamic prediction interval *PrI* for online measurements was calculated by the error-in-variable method [55]:

(17) $$PrI=\hat{y}\pm t_{df,1-\alpha /2}\cdot sde\cdot \sqrt{1+h+n_{cal}^{-1}}$$

where $\hat{y}$ is the predicted value, *t* the critical value from the t-distribution at a significance level *α*, *df* the degrees of freedom (calculated according to the method proposed by van der Voet (1999) [56]), *sde* refers to the estimated standard deviation of the fit error of the calibration data set, *n_cal_* is the number of samples in the calibration data set and *h* refers to the leverages of each spectrum of the online measurement. In contrast to the previous approaches, the leverages *h_ii_* of the *ith* sample were calculated using the general definition

(18) $$h_{ii}={\left[t^{\prime }{\left(T^{\prime }T\right)}^{-1}t\right]}_{ii}$$

in which T refers to the score matrix of the calibration samples and *t* to the score matrix of the samples to predict. Hence, *h_ii_* describes the distance of new samples to samples used for calibration in the x-space. In Figure 7c, the PI shrinks noticeably from roasting time of 7 to 9 min because the predicted Colorette value of 137 exceeds the maximum Colorette value of the calibration (126), which underline the influence of leverages for the prediction. Despite the slightly different roast profile between REMPI at 248 nm and SPI at 118 nm in a previous study, the trends of the predicted Colorette and FC values in a single roast experiment are similar compared to the results from SPI measurements [28]: After an almost linear decrease of the Colorette values, it levels off at darker bean color. In addition, FC values for REMPI at 248 nm show first a slight increase between 7 and 8 min of roast time followed by a steady decrease approaching 2300 GA-eq. mg L^−1^. The different temporal evolutions at the two different REMPI wavelengths are likely caused by the difference in roast profile, but might be also a consequence of the worse quality of calibration for REMPI at 266 nm.

## 4. Conclusions

Resonance-enhanced multi-photon ionization time-of-flight mass spectrometry (REMPI-TOFMS) has been demonstrated as a suitable tool for real-time prediction of bean color and antioxidant capacity by means of Colorette and FC value during coffee bean roasting with a time resolution of 5 s. Based on the extensive characterization of the prediction models, REMPI at 248 nm using an KrF excimer laser show better performances in particular for FC value prediction than REMPI at 266 nm using the fourth harmonic generation from a Nd:YAG laser. Regarding Colorette values, the difference is negligible. However, due to the different ionization selectivity of the two wavelengths, both ionization modes may be of interest for studying the formation or degradation of specific compounds or compound classes in the roast gas.

Comparing REMPI-TOFMS to a previously published explanatory model of the same coffee batch and roast gas analysis by SPI-TOFMS [28], both models for Colorette bean color and FC antioxidant capacity have the lowest RMSE for REMPI at 248 nm (RMSE_CV_ of SPI vs. RMSE_P_ of REMPI). However, for the generation of the SPI model only 20 roast experiments have been included. Hence, the model figure of merit cannot be directly compared and gives only an indication of the potential of SPI-TOFMS for the prediction of Colorette bean color and FC antioxidant capacity. Since SPI ionizes a variety of compounds, which can be assigned to several fundamental processes in coffee roasting, it may be possible to obtain enhanced model performance with a larger data set.

The radiation for ionization was provided by established and robust laser systems with fixed frequencies, so the necessity of tuning sensitive optical devices, such as optical parametric oscillators (OPO) or VUV gas cells [9], could be avoided. Furthermore, the TOFMS works with unit mass resolution, thus dropping mass resolution/mass accuracy or mass shifts from changing or high temperatures does not significantly affect the data quality. Therefore, REMPI-TOFMS is a robust process analytical tool and may be applicable for permanent operation in industrial coffee roasting facilities in a non-temperature-controlled environment [10]. However, for fundamental investigations of the coffee roasting process in laboratory environment, alternating REMPI from a fixed-frequency UV-laser and a laser-pumped OPO for tunable UV-wavelengths might provide detailed insights into dependencies between roasting time and the formation mechanisms of different compound classes.

Ultimately, the combination of SPI and REMPI would unify information about the molecular composition of the roast gas from two complementary techniques, which might lead to a higher selectivity and consequently enhanced description quality for the temporal evolution of coffee bean properties during roasting. A prototype of an SPI/REMPI-TOFMS is developed within this project and experienced in industrial roasting over several hours.

## Figures and Tables

**Table 1:** Figures of merit: Model properties and precision in prediction. Numbers in brackets refer to standard deviation of results from 100 repetitions of competitive adaptive reweighted sampling (CARS).

REMPI Wavelength	Coffee Property	RMSE_CV_	RMSE_P_	Rel. RMSE_P_	R^2^_CV_	R^2^_P_	#LV ^2^	#Var ^3^	RER
248 nm	Colorette	3.88 (0.09)	4.54	0.040	0.985 (0.001)	0.980	6 (1)	16 (2)	23
266 nm	Colorette	4.69 (0.04)	4.63	0.050	0.924 (0.001)	0.926	2 (0)	10 (2)	11
248 nm	FC	94.6 (0.26) ^1^	80.3 ^1^	0.031	0.753 (0.001)	0.822	1 (0)	8 (4)	7.6
266 nm	FC	146 (4.2) ^1^	151 ^1^	0.057	0.490 (0.029)	0.454	2 (0)	4 (2)	4.0

^1^ in GA-eq mg L^−1^, ^2^ #LV: number of latent variables (PLS components), ^3^ Var: number of variables (*m/z*) in PLS regression model.

**Table 2:** Figures of merit: Sensitivity and selectivity.

REMPI Wavelength	Coffee Property	SEN_b_^1^	SEN_NAS_^1^	ASEN ^2^	(ASEN^-1^) ^3^	SEL ^4^
248 nm	Colorette	5.59·10^−4^	7.48·10^−4^	0.305	3.27	8.63
266 nm	Colorette	1.41·10^−3^	8.70·10^−4^	0.359	2.79	16.1
248 nm	FC	3.41·10^−4^	1.21·10^−4^	0.297	3.37	16.3
266 nm	FC	3.7·10^−5^	1.31·10^−5^	0.013	77.7	19.6

^1^ sensitivity in (a.u. L GA-eq mg-1) for FC value and (a.u.) for Colorette value, ^2^ analytical sensitivity in (L GA-eq mg-1) for FC value and [ ] for Colorette value, ^3^ inverse analytical sensitivity in (GA-eq mg L−1) for FC value and (a.u.) for Colorette value, ^4^ selectivity with the unit (%).

**Table 3:** Figures of merit: Limit of detection (LOD).

REMPI Wavelength	Coffee Property	LOD_pu_	LOD_3×RMSEP_	LOD_NAS_	LOD_min_	LOD_max_	LOD_ss_^2^
248 nm	Colorette	6.34	13.6	5.94	2.17	3.12	2.41
266 nm	Colorette	11.6	13.9	10.6	3.91	4.46	3.89
248 nm	FC ^1^	216	241	35.7	127	297	146
266 nm	FC ^1^	487	454	608	138	492	214

^1^ in GA-eq mg L^−1^, ^2^ median value of sample-specific LOD.

**Table 4:** Correlation matrix of target projection (TP) loadings with Pearson’s r in upper and -log_10_(p) in the lower triangular part of the matrix. Significant correlations (α = 0.05, -log_10_(p) > 1.301) are indicated in italic.

**Colorette 248nm**	*0.980*	*0.408*	0.010
*290*	**FC Value 248nm**	*0.383*	0.002
*18.2*	*16.0*	**Colorette 266nm**	*0.598*
0.111	0.024	*41.8*	**FC Value 266nm**

**Figure A1:**
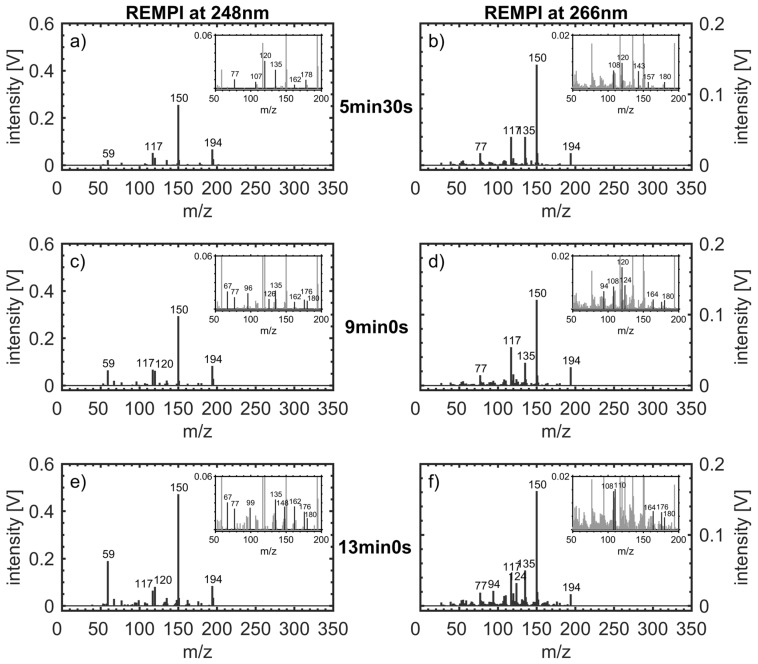
REMPI mass spectra at 248 nm (left) and 266 nm (right) for roasting times 5 min 30 s (**a**,**b**), 9 min (**c**,**d**) and 13 min (**e**,**f**).

**Figure A2:**
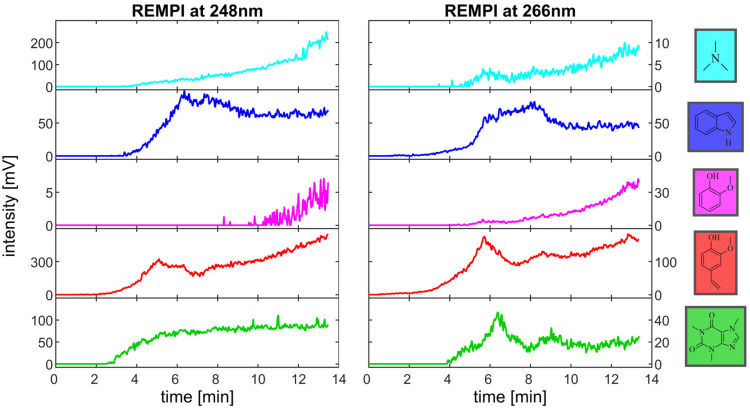
Separated illustration of time traces (1 s time resolution) in Figure 3 for REMPI at 248 nm (left) and REMPI at 266 nm (right) for C3-amine (*m/z* 59; cyan), indole (*m/z* 117; blue), guaiacol (*m/z* 124; magenta), vinylguaiacol (*m/z* 150; red) and caffeine (*m/z* 194; green). Please note the different y-axis scales.

## Appendix A

**Table A1:** List of most abundant ions and structures assigned from Hertz-Schünemann et al. (2013), Czech et al. (2016) and references therein.

*m*/*z*	Structure Assignment		
59	C3-amines	126	hydroxymethylfurfural
66	fragment of phenolic species	131	unknown fragment
67	pyrrole	132	cinnamaldehyde, vinylbenzaldehyde
77	fragment of aromatic species	135	fragment from vinylguiacol
91	fragment of alkylated aromatics	136	vinyldihydroxybenzene
94	phenol	143	unknown fragment
96	furfural	148	2,2’-methylen-bis-furan
99	succinimide, methylthiazole	150	vinylguaiacol
107	fragment of vinylguaiacol	157	unknown fragment
108	methylphenol	162	dihydroxy cinnamaldehyde
109	fragment of guaiacol	164	dimethoxystyrene
110	benzenediol, methylfurfural	176	2,2’-methylen-bis(5-methylfuran)
117	indole	178	difurfurylether
120	phenylacetaldehyde	180	caffeic acid
122	dimethylphenol	194	caffeine, ferulic acid
124	guaiacol, methylctechol		

## Appendix C

The appearance of outliers in the model and their influence was investigated based on Q residues and Hotelling’s T^2^ statistics, also known as influence plot. While Q residues simply describe the accuracy of the prediction (here for the calibration data set), Hotelling’s T^2^ refers to a metric for the influence of a single sample on the model. Because Q residues follow a χ^2^- and Hotelling’s T^2^ an F-distribution, confidence limits can be calculated and used for outlier detection. Here, an outlier sample is regarded as a data point which appear both outside the 95% confidence limits of Q and T^2^, i.e., a sample that is bad represented by the model, but has a high influence on the model parameters.
